# Localized Surface Plasmon Resonance‐Enhanced Photocatalytic Antibacterial of In Situ Sprayed 0D/2D Heterojunction Composite Hydrogel for Treating Diabetic Wound

**DOI:** 10.1002/adhm.202303836

**Published:** 2024-08-26

**Authors:** Zhengao Wang, Wei Li, Youzhun Fan, Cairong Xiao, Zhifeng Shi, Yunbing Chang, Guoyan Liang, Chengli Liu, Zurong Zhu, Peng Yu, Xuebin Yang, Zhiguo Song, Chengyun Ning

**Affiliations:** ^1^ School of Materials Science and Engineering South China University of Technology Guangzhou 510006 P. R. China; ^2^ National Engineering Research Center for Tissue Restoration and Reconstruction South China University of Technology Guangzhou 510006 P. R. China; ^3^ Department of Orthopedics Guangdong Provincial People's Hospital Guangzhou 510080 P.R. China; ^4^ Biomaterials and Tissue Engineering Group School of Dentistry University of Leeds Leeds LS97TF UK; ^5^ School of Materials Science and Engineering Kunming University of Science and Technology Kunming 650093 P.R. China

**Keywords:** antibacterial, diabetic wounds, heterojunction, localized surface plasmon resonance, NIR‐activable

## Abstract

Chronic diabetic wounds pose significant challenges due to uncontrolled bacterial infections, prolonged inflammation, and impaired angiogenesis. The rapid advancement of photo‐responsive antibacterial therapy shows promise in addressing these complex issues, particularly utilizing 2D heterojunction materials, which offer unique properties. Herein, an in situ sprayed Bi/BiOCl 0D/2D heterojunction composite fibrin gel with the characteristics of rapid formation and effective near‐infrared activation is designed for the treatment of non‐healing diabetes‐infected wounds. The sprayed composite gel can provide protective shielding for skin tissues and promote endothelial cell proliferation, vascularization, and angiogenesis. The Bi/BiOCl 0D/2D heterojunction, with its localized surface plasmon resonance (LSPR), can overcome the wide bandgap limitation of BiOCl, enhancing the generation of local heat and reactive oxygen species under near‐infrared irradiation. This facilitates bacterial elimination and reduced inflammation, supporting the accelerated healing of diabetes‐infected wounds. This study underscores the potential of LSPR‐enhanced heterojunctions as advanced wound therapies for chronic diabetic wounds.

## Graphical abstract

A smart Bi/BiOCl 0D/2D heterojunction‐based in situ sprayed fibrin gel with the characteristics of rapid formation and effective near‐infrared activation is designed. This innovative gel accelerates healing by promoting angiogenesis, eliminating bacteria, and reducing inflammation. It effectively addresses the challenges of chronic diabetic wounds, offering a promising solution for improved wound therapy.

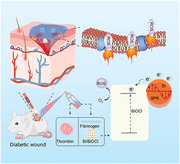

## 1 Introduction

Non‐healing diabetic wounds are a chronic complication of diabetes mellitus, posing significant threats to patients' health and quality of life.^[^ ^1^ ^]^ The management of chronic diabetic wounds presents substantial challenges, including the risks of uncontrolled bacterial infection, long‐term inflammation, and impaired angiogenesis, leading to compromised oxygen delivery.^[^ ^2^ ^]^ Accelerating angiogenesis, promoting local neovascularization and peripheral blood flow, on‐demand sterilization, and reducing inflammation in diabetic wounds are crucial for facilitating chronic wound healing.^[^ ^3^ ^]^ In traditional clinical practice, diabetic wounds are treated with infection control, wound debridement, graft transplantation, blood glucose management, and early graft revascularization.^[^ ^4^ ^]^ However, these strategies only provide limited wound healing outcomes. Although wound autografts, novel dressings, and tissue engineering products have shown potential in promoting vascularization in diabetic wounds, the complex microenvironment of diabetic wounds and the lack of coordinated treatment design have hindered their full effectiveness.^[^ ^5^ ^]^ Therefore, there is an urgent need to develop advanced and effective wound therapies that can facilitate angiogenesis, neo‐vascularization, and timely resolution of inflammation and bacterial infections in chronic diabetic wound tissues.

With the rapid development of nanotechnology, photo‐responsive antibacterial therapy has emerged as a promising alternative for diabetic wound treatment.^[^ ^6^ ^]^ This approach offers non‐invasiveness and a broad antibacterial spectrum through redox reactions at the nano–bio interface.^[^ ^7^ ^]^ Specifically, bismuth oxychloride (BiOCl), a 2D material, in conjunction with van der Waals heterojunctions, exhibits remarkable potential as a photocatalytic antibacterial agent.^[^ ^8^ ^]^ This is attributed to its distinctive physicochemical properties, such as an exceptionally large specific surface area, adjustable electronic structure, minimal electron/hole recombination rates, and a wealth of surface‐active sites.^[^ ^8^, ^9^ ^]^ These properties surpass the limitations of traditional lattice‐matching requirements. Unfortunately, the intrinsic wide bandgap of BiOCl limits its application to short‐wavelength range light, leading to suboptimal photocatalytic performance in facilitating reactive oxygen species (ROS) generation.^[^ ^10^ ^]^ Various methods, such as heteroatom doping,^[^ ^11^ ^]^ defect formation,^[^ ^12^ ^]^ and heterostructure design,^[^ ^13^ ^]^ have been explored to improve light response. Among these methods, localized surface plasmon resonance (LSPR) has emerged as a favorable solution to enhance light absorption, inhibit photogenerated charge carrier recombination, and improve the photocatalytic antibacterial performance of BiOCl.^[^ ^14^ ^]^ LSPR occurs on the surface of metal nanostructures, such as gold,^[^ ^15^ ^]^ silver,^[^ ^16^ ^]^ copper,^[^ ^17^ ^]^ and bismuth,^[^ ^18^ ^]^ and the formation of heterojunctions by coupling plasmonic nanostructures with semiconductors holds great potential for antibacterial phototherapy via plasmonic energy transfer from the metal nanostructure to the semiconductor.^[^ ^19^ ^]^

**Figure 1:**
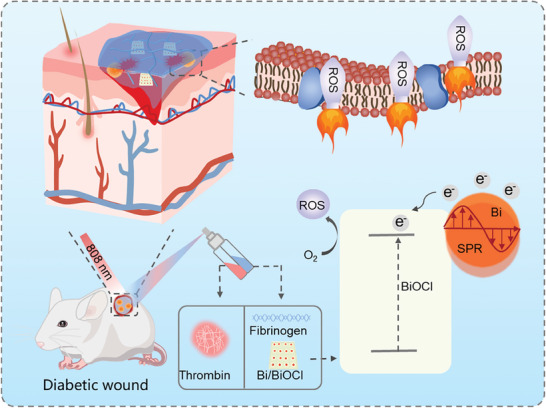
Schematic illustration of Bi/BiOCl nano‐heterojunction‐based in situ sprayed composite hydrogel for LSPR‐enhanced photocatalysis antibacterial, facilitating angiogenesis and accelerating diabetic wound healing.

In this study, an in situ sprayed Bi/BiOCl 2D/0D heterojunction composite hydrogel had been developed. It had an LSPR‐enhanced antibacterial effect for the treatment of diabetes‐infected wounds. The Bi/BiOCl heterojunction was synthesized via an in situ reduction method. In addition, the Bi/BiOCl was incorporated into fibrin gel, which had unique merits including good biocompatibility,^[^ ^20^ ^]^ amenability to a simple spray‐coating approach,^[^ ^21^ ^]^ and wound healing through promoting angiogenesis.^[^ ^22^ ^]^ The Bi/BiOCl heterojunction with LSPR could effectively promote NIR absorption, leading to enhanced antibacterial efficacy. Therefore, the engineered Bi/BiOCl heterojunction composite hydrogel not only temporarily shielded tissues from the external environment and promoted endothelial cell proliferation and vascularization but could also generate local heat and ROS under near‐infrared light laser (NIR) (808 nm) irradiation to eliminate bacteria and reduce inflammation (**Figure** **1**). Herein, we provided the proof‐of‐principle evidence for the use of an in situ sprayed NIR‐activated Bi/BiOCl heterojunction composite hydrogel to support accelerated diabetes‐infected wound healing.

## 2 Results and Discussion

**Figure 2:**
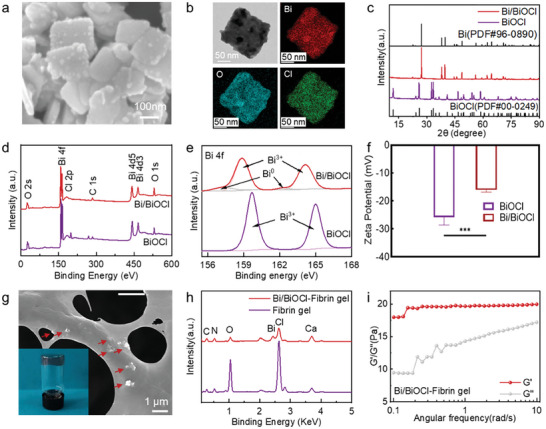
The preparation and characterizations of Bi/BiOCl@Fibrin gel. a) FE‐SEM images of BiOCl and Bi/BiOCl. b) TEM of Bi/BiOCl and elemental mapping images of Bi, O, and Cl. c) XRD patterns of Bi/BiOCl. d) XPS survey spectra of BiOCl and Bi/BiOCl. e) XPS narrow scan for Bi. f) Zeta potential of BiOCl and Bi/BiOCl. g) FE‐SEM images of Bi/BiOCl@Fibrin gel. h) EDS spectrum of Bi/BiOCl@Fibrin gel and Fibrin gel. i) Rheological properties of Bi/BiOCl@Fibrin gel and Fibrin gel. Data for (f) represents mean +/− SD. *n* = 3. ***P < 0.001.

NIR‐activable Bi/BiOCl heterojunctions were facilely synthesized via an in situ reduction method. The BiOCl nanosheets exhibited a square 2D structure with a width of 100–300 nm, and Bi nanoparticles were in situ grown on the surface of BiOCl, as shown in **Figure** **2**a,b. The density and size of Bi nanoparticles could be controlled by tuning the concentration of NaBH_4_ (Figure S1, Supporting Information). TEM images of Bi/BiOCl revealed uniform dispersion of Bi nanoparticles with a width of 10–30 nm on the BiOCl nanosheet surfaces. The high‐resolution TEM (HRTEM) image in Figure S2b, Supporting Information exhibits well‐crystallized BiOCl nanosheet with clear lattice fringes, featuring a *d*_110_ spacing of 0.275 nm. In addition, the HRTEM image suggests the possible presence of subgrains within the BiOCl nanosheet. The selected area electron diffraction (SAED) pattern from one of the BiOCl nanosheet displayed sharp diffraction spots indicative of good crystallization (Figure S2c, Supporting Information). However, the irregular and non‐periodic arrangement of diffraction spots supported the potential existence of subgrains in the BiOCl nanosheet. In the case of the Bi/BiOCl sample, HRTEM image (Figure S2e, Supporting Information) revealed that some BiOCl nanosheets were reduced by NaBH_4_ to form Bi nanoparticles. These Bi nanoparticles exhibited distinct lattice fringes, characteristic of rhombohedrally structured Bi. Further, minor amorphous Bi was observed, particularly at the surface of Bi crystals, as indicated by the HRTEM images. This observation was corroborated by the SAED patterns of Bi/BiOCl (Figure S2f, Supporting Information), which exhibited diffuse diffraction rings typical of an amorphous material.

XRD analysis (Figure 2c) confirmed the diffraction peaks of the 2D nanosheet belonging to the BiOCl phase. In addition, new diffraction peaks at 2*θ* values of 27.1°, 37.9°, and 39.6° corresponded to the metallic Bi (PDF#00‐0249).^[^ ^23^ ^]^ Figure S3, Supporting Information illustrates the XRD patterns of pure BiOCl and NaBH_4_‐treated BiOCl samples with varying concentrations. Upon treatment with NaBH_4_ solution, additional diffraction peaks corresponding to metallic Bi were observed in the XRD patterns. Notably, the intensity of the metallic Bi diffraction peaks gradually increased with increasing concentration of NaBH_4_ solution. When the NaBH_4_ solution concentration reached 100 mm, a pure metallic Bi phase was formed. Moreover, the partial magnified detail of 100Bi/BiOCl suggested the persistence of the crystal structure of BiOCl. These XRD results suggested that NaBH_4_ treatment induced the progressive reduction of BiOCl into metallic Bi. The XPS survey spectrum (Figure 2d) confirmed the main elements in Bi/BiOCl as Bi, O, and Cl. The peaks at 165.0 and 159.2 eV were attributed to Bi^3+^ 4f; while, the peaks at 162.3 and 157.0 eV originated from Bi^0^ 4f, indicating partial reduction of BiOCl to metallic Bi^[^ ^24^ ^]^ (Figure 2e). Further XPS narrow scan analysis for O corroborated that NaBH_4_‐mediated reduction could enhance oxygen vacancies (Figure S4a, Supporting Information). The red‐shift in banding energy of Bi^3+^ and O suggested that electrons in the Bi nanoparticle could transfer to Bi^3+^ and the oxygen vacancy state, consistent with the results of density‐functional theory (DFT) calculations. To delve deeper into the role of oxygen vacancies in the redox process of Bi^3+^, detailed electron spin resonance (ESR) analysis was employed. As depicted in Figure S4b, Supporting Information, testing the pure BiOCl sample revealed the presence of a characteristic peak, indicating the existence of oxygen vacancies. Upon addition of NaBH_4_, the strength of this characteristic peak in the synthesized compound notably increased. Moreover, the average zeta potential of nanoparticles changed from −25.8 mV for BiOCl to −16.05 mV for Bi/BiOCl, confirming the strong electrostatic adsorption of Bi nanoparticles on BiOCl^[^ ^25^ ^]^ (Figure 2f).

Bi/BiOCl nanosheets were incorporated into the fibrin gel to fabricate nanocomposite hydrogels. A vial turnover test confirmed the successful formation of the Bi/BiOCl@Fibrin gel (inset in Figure 2g). SEM images displayed uniform distribution of Bi/BiOCl within the fibrin network structure of the hydrogel (Figure 2g). EDS analysis confirmed the presence of Bi, O, and Cl elements in the Bi/BiOCl@Fibrin gel (Figure 2h). Appropriate rheological properties are crucial for the spray forming operation of the hydrogel, which is essential for effective wound treatment. The rheological properties of the Bi/BiOCl@Fibrin gel were subsequently evaluated. Throughout the entire angular frequency range, the storage modulus (*G*′) of the Bi/BiOCl@Fibrin gel consistently exceeded the loss modulus (*G*″), indicative of its hydrogel characteristics^[^ ^26^ ^]^ (Figure 2i). In addition, investigation of the rheological properties of the Bi/BiOCl@Fibrin gel with varying concentrations of Bi/BiOCl heterojunction showed no significant impact on the gel's characteristics (Figure S5, Supporting Information). These findings demonstrate the successful incorporation of Bi/BiOCl nanosheets into the fibrin gel matrix to form nanocomposite hydrogels. The resulting Bi/BiOCl@Fibrin gel exhibited favorable rheological properties, which holds promise for various biomedical applications.

**Figure 3:**
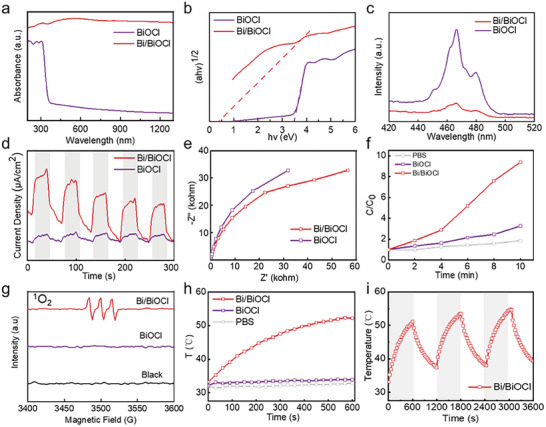
NIR‐excited electro/hole separation, ROS generation, and LSPR‐induced photothermal of the Bi/BiOCl nanojunction. a) UV–vis–NIR absorption spectrum, b) the plot of α*h*ν^1/2^ versus *hv*, c) PL spectrum, d) photocurrent, and e) electrochemical impedance spectroscopy of BiOCl and Bi/BiOCl. f) Bi/BiOCl‐induced ROS production. g) Singlet oxygen in an aqueous dispersion with TEMP being irradiated with NIR. h) Photothermal heating curves and i) cyclic photothermal heating curves of BiOCl and Bi/BiOCl.

A comprehensive investigation was conducted to look at the optical absorption and near‐infrared (NIR)‐excited electro/hole separation of Bi/BiOCl heterojunctions. UV–vis diffuse reflectance spectroscopy revealed that BiOCl solely absorbed UV light, whereas Bi/BiOCl exhibited a continuous and robust absorption across the range of 200–1300 nm (**Figure** **3**a). Utilizing the Kubelka–Munk function,^[^ ^27^ ^]^ we calculated the band gap values for BiOCl and Bi/BiOCl. The band gap of Bi/BiOCl was found to be substantially reduced from 3.48 to 0.40 eV compared to pure BiOCl (Figure 3b; Figure S6, Supporting Information). In addition, the significant overlap between the UV–vis absorption of Bi/BiOCl and LSPR band of Bi may potentially interfere with the determination of the band gap. To investigate the recombination rate of free electrons and holes, we performed fluorescence analysis (Figure 3c). Two characteristic bands in the fluorescence spectra at 465 and 479 nm corresponded to direct and indirect electron–hole recombination, respectively.^[^ ^28^ ^]^ Remarkably, the characteristic bands of the Bi/BiOCl heterojunctions were barely discernible, indicating significantly lower charge recombination compared to pure BiOCl. The PL emission intensity decreased with the density of Bi nanoparticle, indicating that the photoinduced dissociated electrons were rapidly transferred from Bi nanoparticle to BiOCl, leading to PL quenching, and effectively, suppressing the recombination of photogenerated carriers in Bi/BiOCl nanoheterojunction (Figure S7, Supporting Information).^[^ ^29^ ^]^ The photocurrent response was evaluated under several switching cycles of intermittent NIR to assess charge separation efficiency. The Bi/BiOCl photocurrent response was five times stronger than that of BiOCl, demonstrating that Bi/BiOCl exhibited higher charge separation efficiency than pure BiOCl (Figure 3d).^[^ ^30^ ^]^ In addition, we used electrochemical impedance spectroscopy (EIS) to analyze electron transfer at the interface. The EIS of Bi/BiOCl exhibited a smaller semicircle size, suggesting lower electrical resistance compared to BiOCl (Figure 3e).

Quantitative analysis of reactive oxygen species (ROS) generation induced by pure BiOCl and Bi/BiOCl was performed using the non‐specific reactive oxygen detection probe 2′,7′‐Dichlorodihydrofluorescein (DCFH). The DCFH was oxidized to form DCF by ROS, and the characterization of DCF by a microplate reader confirmed ROS generation. As shown in Figure 3f; Figure S8, Supporting Information, when near‐infrared light was irradiated for 10 min, the control group produced negligible ROS; while, the ROS production of the Bi/BiOCl heterojunction gradually increased. Among them, 100Bi/BiOCl could be increased to ≈ten times. To discern ROS species with precision, including superoxide radical (·O_2_^−^), singlet oxygen (^1^O_2_), and hydroxyl radicals (·OH), we employed ESR spin trapping with spin labeling. The solutions containing Bi/BiOCl or BiOCl samples and spin probes were irradiated by NIR for 10 min. The Bi/BiOCl sample displayed a typical ESR spectrum with three lines having relative intensities of 1:1:1; while, the pure BiOCl sample showed no characteristic ESR peaks (Figure 3g). This result demonstrated that Bi/BiOCl could catalyze the generation of singlet oxygen (^1^O_2_) under NIR irradiation owing to the localized surface plasmon resonance (LSPR) enhancement of Bi nanoparticles. Further, the amplifying effect of Bi on ·O_2_^−^ generation closely paralleled its impact on ^1^O_2_ generation (Figure S9a, Supporting Information). It is noteworthy that neither BiOCl nor Bi/BiOCl could induce ·OH generation due to inherent structural constraints (Figure S9b, Supporting Information). The nanoheterojunction and oxygen vacancies could improve the efficiency of photogenerated charge separation and enhance O_2_ adsorption performance of BiOCl. The photo‐generated electrons could combine with O_2_ adsorbed on the surface of catalyst to form superoxide radicals (·O_2_^−^), which could be further oxidized to singlet oxygen (^1^O_2_).^[^ ^31^ ^]^ Overall, these findings indicate that the Bi/BiOCl heterojunctions possessed enhanced optical absorption, improved charge separation efficiency, and superior ROS generation capabilities under NIR excitation compared to pure BiOCl.

As depicted in Figure 3h, the Bi/BiOCl heterojunctions exhibited rapid temperature elevation from 32.0 °C to 51.0 °C within 10 min upon exposure to NIR light. This significant increase in temperature was attributed to the localized surface plasmon resonance effects of Bi nanoparticles integrated into the heterojunction. In contrast, pure BiOCl showed almost negligible change in surface temperature under the same NIR irradiation conditions. Moreover, the laser on–off cycles of Bi/BiOCl, as depicted in Figure 3i, demonstrated that repeated irradiation had minimal influence on the photothermal properties, highlighting the exceptional photostability of Bi/BiOCl. These remarkable findings collectively signify the superior NIR activable activity of the Bi/BiOCl heterojunctions.

**Figure 4:**
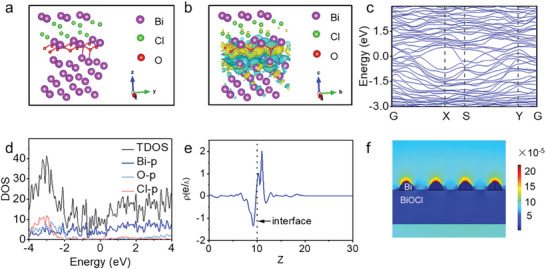
The density‐functional theory (DFT) calculations and the electromagnetic field distribution of Bi/BiOCl. a) The simulated crystallographic structure and b) differential charge density map of Bi/BiOCl. The green and yellow areas represent the decreased electron density (electron loss area) and increased electron density (electron area), respectively. c) Band structure, d) density of states (DOS), and e) plane‐averaged differential charge density of Bi/BiOCl based Vienna ab initio simulation package (VASP). f) The electromagnetic field distribution of Bi/BiOCl by COMSOL Multiphysics.

The density functional theory calculations revealed that the Bi atom of Bi nanoparticle loses electrons and BiOCl gains electrons, resulting in electrons transferring from Bi nanoparticle to BiOCl at the interface of Bi/BiOCl (**Figure** **4**a,b).^[^ ^32^ ^]^ According to the calculation results of energy bands, it can be concluded that Bi/BiOCl interfaces exhibited metallic characteristics (Figure 4c). The metal–semiconductor heterojunction interfaces favored charge separation and transferred once they were excited. The density of states (DOS) further proved that the interface of Bi/BiOCl exhibited metallic characteristics, and the electronic states near the Fermi level were mainly occupied by Bi‐p orbitals (Figure 4d). The plane‐averaged differential charge density showed that at the interface of Bi/BiOCl, Bi atom of Bi nanoparticle lost electrons, BiOCl gained electrons, and electrons transferred from Bi to BiOCl (Figure 4e).^[^ ^33^ ^]^ To demonstrate the LSPR enhancement of the Bi nanoparticle, we simulated the electromagnetic field distribution on the surface of Bi/BiOCl heterojunctions based on COMSOL finite element analysis. As shown in Figure 4f, the electromagnetic field was greatly enhanced at the top of Bi nanoparticle under NIR irradiation. The LSPR effect of Bi nanoparticle generated a concentrated “hot spot” region to generate many photoelectrons; and then, the excited electrons were quickly transferred to the surface of BiOCl by the built‐in electric field created by Bi nanoparticle and BiOCl. In addition, given its large surface area, BiOCl provided considerably active sites for ROS generation. This would lead to less recombination and fast separation/collection processes, which would agree well with the above photoluminescence spectra.

**Figure 5:**
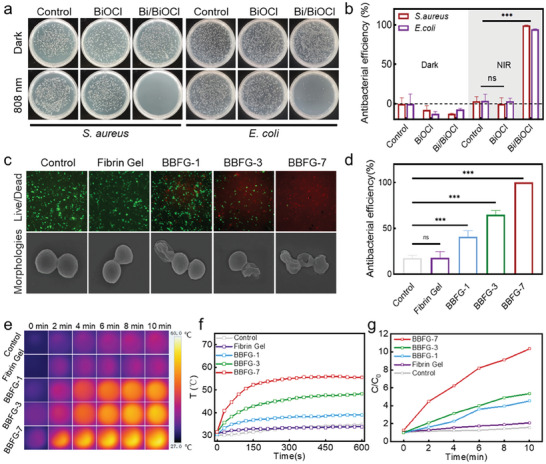
Antibacterial properties of the Bi/BiOCl@Fibrin gel. a) Photographs of bacterial colonies formed by *S. aureus* and of *E. coli*. After treatment with BiOCl or Bi/BiOCl without or with 808 nm irradiation. b) Antibacterial efficiency of Bi/BiOCl against *S. aureus* and or *E. coli*. c) SEM images and live/dead fluorescent photos through calcein‐AM/PI staining of *S. aureus*. d) Antibacterial efficiency of Bi/BiOCl@Fibrin gel against *S. aureus*. e) Real‐time infrared thermal images and f) photothermal heating curves of pure fibrin gel and Bi/BiOCl nanocomposite gel. g) ROS production of Bi/BiOCl nanocomposite gel under 808 nm irradiation as determined by DCFH. Data for (b,d) represents Mean +/− SD. *n* = 3. ns, not statistically significant. ***P < 0.001.

Bacterial infection treatment is critical for chronic wound tissue of Diabetes Mellitus in clinical practice. Based on the promising NIR‐responsive activity of Bi/BiOCl heterojunctions, engineered Bi/BiOCl@Fibrin gel could prevent wound infections. First, we evaluated the antibacterial effect of Bi/BiOCl heterojunction in vitro under the NIR irradiation. The spread plate result (**Figure** **5**a) revealed that the Bi/BiOCl exerted negligible effects on bacterial viability in the dark. By contrast, Bi/BiOCl exhibited promising antibacterial activity against *Staphylococcus aureus* and *Escherichia coli* under the NIR irradiation. As shown in Figure 5b, the inactivation rate of *S. aureus* and *E. coli* reached 99.38% and 94.58%, respectively. At the same time, the antibacterial rate increased with the density of bismuth particles (Figure S10, Supporting Information). We further assessed minimum inhibitory concentrations (MICs) of photo‐responsive Bi/BiOCl against methicillin‐resistant *S. aureus* (MRSA) to determine the optimal concentration for practical applications. The results suggested that Bi/BiOCl at 256–512 µg mL^−1^ was significantly bactericidal (Figure S11, Supporting Information). Thus, we believe that a concentration range of 256–512 µg mL^−1^ is appropriate for practical applications. Further, the recurrence of drug resistance to Bi/BiOCl was evaluated by incubating MRSA with Bi/BiOCl from Passage 1 through multiple growth passages (Passages 2–10). The MICs of Bi/BiOCl against MRSA were determined for each passage to assess the potential for recurrent bacterial resistance. Remarkably, Bi/BiOCl maintained consistent MIC values against MRSA across all ten passages, indicating a minimal risk of acquiring drug resistance over prolonged exposure periods. The excellent antibacterial performance of the Bi/BiOCl heterojunctions inspired us to investigate the antibacterial activity of Bi/BiOCl nanocomposite gel. *S. aureus* was selected as the model bacterium to study the NIR activable antibacterial activity of Bi/BiOCl@Fibrin gel. As expected, Bi/BiOCl@Fibrin gel had a definite antibacterial property upon NIR irradiation. The antibacterial rate of BBFG‐7 (the mass ratio of Bi/BiOCl in the recomposite was 7%) was 99.50% (Figure 5d).

To further verify the antibacterial ability, live/dead staining assay and SEM were employed to examine structural integrity of bacterial membranes. As shown in Figure 5c, live bacteria with intact cytoplasmic membranes were stained by membrane‐permeable SYTO9 (green); while, dead bacteria with degraded cytoplasmic membranes were stained by membrane‐impermeable PI (red). There was hardly any red fluorescence observed in the control group, indicating the integrity of bacterial membrane structure. In addition, the Bi/BiOCl@Fibrin gel group displayed enhanced red fluorescence and bacteria lost their membrane integrity. Afterward, the integrity of the bacterial membrane was examined by SEM (Figure 5c). The normal *S. aureus* showed an intact spherical structure with glossy and unharmed membranes. After treatment with Bi/BiOCl@Fibrin gel and NIR irradiation, the *S. aureus* cytoplasmic membranes were seriously compromised with obvious surface collapse, which was consistent with the live/dead staining assay.

We assessed the photothermal performance of the Bi/BiOCl nanocomposite gel using real‐time infrared thermal images and photothermal heating curves, with irradiation from 808 nm near‐infrared light. The temperature of gels containing varying amounts of Bi/BiOCl heterojunction gradually increased (Figure 5f), and the hydrogels with 7% Bi/BiOCl exhibited the most significant heating effect, reaching a temperature increase of 25 °C within 200 s under a power density of 1.5 W cm^−2^. In contrast, the pure fibrin gel did not show a noticeable temperature increase, indicating that the hydrogel gained photothermal properties after the addition of Bi/BiOCl. To evaluate the production of reactive oxygen species (ROS), DCFH was employed. As shown in Figure 5g, under 808 nm irradiation, no ROS production was detected in the control group or the fibrin gel group. However, the BBFG‐7 gel demonstrated ≈ten times the ROS production within 10 min. These results validate the strong NIR responsiveness of the Bi/BiOCl nanocomposite gel and highlight its potential for NIR‐responsive therapy in antimicrobial applications.

**Figure 6:**
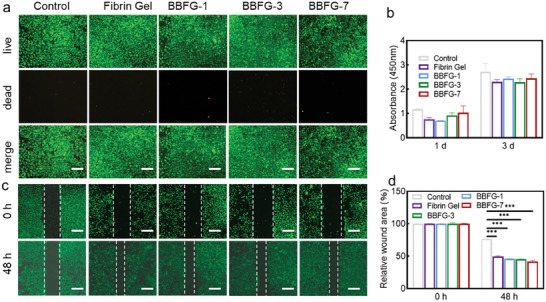
Biocompatibility evaluation of Bi/BiOCl@Fibrin gel in vitro. a) Live/dead staining of HUVEC cells after 24 h of contact with different samples. Green fluorescence indicates live cells; while, red fluorescence indicates dead cells (The scale bar is 200 µm). b) Relative absorbance at 450 nm of HUVEC cells after 1 and 3 days of contact with different samples. c) Calcein AM stained imaging showing the migration of HUVECs at different time points (The scale bar is 500 µm). d) Quantitative analysis of HUVEC migration, represented as the percentage of wound closure over time. The number of samples for (a–d) is *n* = 3. ***P < 0.001.

It can be hypothesized that the Fibrin gel, composed of thrombin and fibrinogen, would exhibit biocompatibility as it could serve as a scaffold for cell proliferation. To evaluate this, live/dead cell staining and CCK‐8 assays were performed on HUVEC cells. As depicted in **Figure** **6**a, HUVEC cells incubated within the Bi/BiOCl@Fibrin gel demonstrated a high cell density and a homogeneous cell morphology, exhibiting robust green fluorescence indicative of live cells. Conversely, there was a minimal presence of dead cells, suggesting that the gel provided a favorable microenvironment conducive to cell viability and survival. Consistent with the live/dead staining results, the CCK‐8 cell cytotoxicity assay (Figure 6b) corroborated the excellent biocompatibility of the Bi/BiOCl@Fibrin gel. The assay demonstrated a high cell viability, indicating that the gel did not elicit significant cytotoxic effects or impede cell growth and proliferation. To further elucidate the effect of the Bi/BiOCl@Fibrin gel on cell migration, we employed a scratch assay. A controlled scratch was created on a 24‐well plate, followed by the placement of transwells containing the hydrogel scaffolds to assess HUVEC cell migration. After a 48‐h incubation period, remarkable cell migration and wound closure were observed in the Bi/BiOCl@Fibrin gel group (Figure 6c). The wound area in the test group exhibited a substantial reduction to 40.16% compared to the initial wound size. In contrast, the negative control group displayed a less pronounced wound closure, with 76.67% of the wound area remaining (Figure 6d). These findings suggested that Bi/BiOCl@Fibrin gel exhibited high biocompatibility and had the potential to promote wound healing and skin regeneration. These results highlight the promising application of Bi/BiOCl@Fibrin gel in the field of wound healing and skin regeneration, demonstrating its ability to support cell viability, migration, and tissue repair.

**Figure 7:**
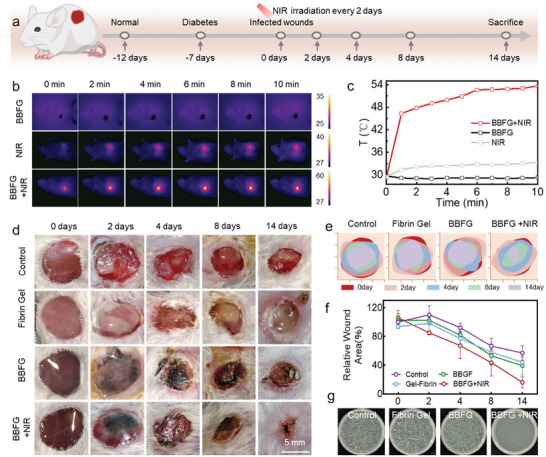
Bi/BiOCl@Fibrin gel accelerated wound healing. a) The animal model of *S. aureus*‐infected diabetic wounds. b) Real‐time infrared thermal images showing temperature changes in the treated wounds. c) Photothermal heating curves depicting the temperature rise in mice with different treatments. d) Sequential photographs of skin wounds at days 0, 2, 4, 8, and 14 in different experimental groups. e) Wound healing boundaries at different time points in the various groups. The red area represents the initial wound area; while, the pink, blue, green, and purple areas represent the wound area on days 2, 4, 8, and 14, respectively. f) Quantitative analysis of the wound areas at different time points, demonstrating the progression of wound healing. g) Digital photographs showing *S. aureus* colonies grown on LB agar plates extracted from different experimental groups.

To evaluate the therapeutic potential of Bi/BiOCl@Fibrin gel in NIR‐activatable antibacterial effects, we established an animal model of *S. aureus*‐infected diabetic wounds in vivo to investigate the wound closure efficacy of the composite hydrogel (**Figure** **7**a).^[^ ^34^ ^]^ The process of diabetic rat modeling involved administering streptozotocin injections for 12 consecutive days. This led to a significant increase in glucose levels, reaching 28.4 mmol mL^−1^ (>16.7), confirming the induction of diabetes in the mice (Figure S12, Supporting Information). The diabetic mice were categorized into four groups: Control group, Fibrin gel group, 7% Bi/BiOCl@Fibrin gel (BBFG) group, and 7% Bi/BiOCl@Fibrin gel + 808 nm for 10 min (BBFG+NIR) group. An infrared thermal camera was utilized to monitor the temperature changes in the BBFG + NIR group, induced by the 808 nm NIR laser (Figure 7b). As depicted in Figure 7c, the temperature of the BBFG + NIR group progressively increased from 30 °C to 53 °C within 6 min. This observed phenomenon aligned with the results of in vitro photothermal experiments and could be attributed to the LSPR effect induced by the Bi/BiOCl heterojunction. Conversely, the control group displayed no significant temperature alteration, thereby further confirming the exceptional NIR‐responsive performance of the composite hydrogel. Representative images of the wounds at predetermined time intervals were captured and presented in Figure 7d, and the wound areas were quantified accordingly (Figure 7e). Notably, the wounds treated with the BBFG+NIR hydrogel exhibited superior healing outcomes compared to the control group, fibrin gel group, and BBFG group (Figure 7f). Specifically, by day 8, the BBFG + NIR group achieved a remarkable 56.53% wound closure, which was significantly higher than the closure rates observed in the control group (33.38%), fibrin gel group (41.93%), and BBFG group (46.91%). Remarkably, by day 14, the wounds treated with BBFG + NIR demonstrated the most advanced healing progress, with only 16.06% of the initial wound area remaining, indicating substantial formation of new skin (Figure 7e,f). To assess the antibacterial efficacy in vivo, bacteria were extracted from the wound tissue and cultured on agar medium for colony counting (Figure 7g; Figure S13, Supporting Information). The results were consistent with the in vitro antibacterial findings, demonstrating that the BBFG + NIR group effectively inhibited bacterial survival and reproduction within the wound area under NIR irradiation. These comprehensive results validated that the treatment utilizing Bi/BiOCl@Fibrin gel, in combination with NIR irradiation, significantly accelerated the healing of diabetic wounds in vivo, particularly in the presence of *S. aureus* infection. The observed enhanced wound closure, promotion of new skin formation, and potent antibacterial effects exhibited by the BBFG + NIR group highlight the promising potential of this approach for the effective management of diabetic wounds. In situ sprayed hydrogels may exhibit low mechanical strength and stability, which limit their practical applications, particularly in outdoor settings or in dynamic environments. To enhance the mechanical properties and stability of the hydrogels, strategies such as crosslinking can be employed to improve their structural integrity and resistance to mechanical stress.

**Figure 8:**
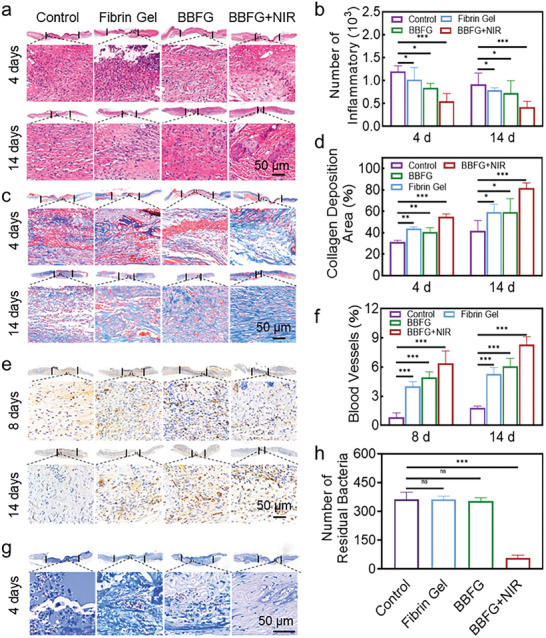
Bi/BiOCl@Fibrin gel reduces inflammation, increases collagen deposition, promotes angiogenesis, and eliminates bacteria in diabetic mice. a) Hematoxylin and eosin (H&E) staining image of mice wound healing at days 4 and 14 with different treatments, highlighting the histological changes. b) Quantitative analysis of inflammatory cells in the wounds. c) Masson's trichrome staining of the wound sections at days 4 and 14, visualizing collagen deposition. d) Quantitative analysis of collagen deposition area. e) Immunohistochemical staining for CD31 in wound tissues at days 8 and 14, indicating the formation of blood vessels. f) Quantification of blood vessel area, providing numerical data on the extent of angiogenesis. g) Giemsa staining of wound tissues after 4 days, revealing the presence of bacteria within the wounds. h) Quantification of residual bacteria in the wounds. Data represent mean +/− SD. *n* = 6. ns, not statistically significant. *P < 0.05, **P < 0.01, and ***P < 0.001.

Diabetic wound healing is a complex and dynamic process involving various biological phases. To provide a comprehensive assessment of the therapeutic effects, histological analysis was conducted to examine the infected tissues of mice treated with the composite hydrogel. Hematoxylin and eosin (H&E) staining (**Figure** **8**a,b) and Masson's trichrome staining (Figure 8c,d) were employed to evaluate the infected tissues of mice. On day 4, the diabetic‐infected wounds exhibited an abundance of inflammatory cells and a loss of collagen fibers. In comparison, the BBFG + NIR group demonstrated a reduced presence of inflammatory cells compared to the other treatment groups. By day 14, in the control group, although collagen fibers had increased, the presence of inflammatory cells persisted, and collagen distribution remained uneven, resulting in delayed wound healing. In contrast, the BBFG + NIR group exhibited a significant reduction in inflammatory cells, accompanied by a well‐organized deposition of collagen, covering 81.81% of the wound area. The anti‐inflammatory nature of BBFG + NIR could be attributed to the bactericidal and immunomodulatory. Fibrin may be a key switch in regulating macrophage phenotype behavior, and this feature may provide a valuable immunomodulatory strategy for tissue healing and regeneration.^[^ ^35^ ^]^ These findings indicated that the BBFG + NIR treatment promoted a more favorable wound‐healing environment characterized by reduced inflammation and enhanced collagen synthesis and deposition.

Neovascularization is a critical process in tissue regeneration, and inadequate local blood supply can impede wound healing.^[^ ^36^ ^]^ To evaluate the formation of microvessels, immunohistochemical staining of CD31 was performed. As depicted in Figure 7e,f, on day 8, all the hydrogel‐treated groups exhibited a limited number of vessels with sporadic distribution of capillaries. Over time, microvessel formation gradually increased. Notably, the BBFG + NIR group demonstrated a significantly higher level of neovascularization compared to the control group by day 14. Fibrin is a major component of the provisional extracellular matrix formed during tissue repair following injury and enables cell infiltration and anchoring at the wound site.^[^ ^37^ ^]^ This suggested that the BBFG + NIR treatment promoted angiogenesis, facilitating improved blood supply to the wound area and supporting tissue regeneration. Further, bacterial infection is a common complication in diabetic wounds and can hinder the healing process. To assess the bacterial load in different groups, Giemsa staining was performed. Figure 7g,h reveals that on day 4, the control group, fibrin gel group, and Bi/BiOCl Fibrin gel group exhibited a substantial presence of bacteria within the wounds. In contrast, the BBFG + NIR group showed minimal bacterial staining, indicating effective antibacterial performance mediated by LSPR effect of the Bi/BiOCl heterojunction. Collectively, the in situ sprayed NIR‐responsive Bi/BiOCl‐based gel in our study demonstrated accelerated wound healing, enhanced angiogenesis, bacterial elimination, and reduced inflammation in diabetic mice.

## 3 Conclusion

Herein, we have successfully developed a novel antibacterial Bi/BiOCl@Fibrin gel with excellent NIR‐responsive antibacterial activity and a high biocompatibility via a super‐convenient strategy. Both in vitro and in vivo results demonstrated that this Bi/BiOCl‐based gel simultaneously and effectively addressed features of the diabetic infected‐wound healing milieu such as chronic wounds, impaired angiogenesis, bacterial infection, and exacerbated inflammation, thereby promising a better therapeutic material for the diabetic wound treatments.

## 4 Experimental Section

### Materials

Bismuth nitrate pentahydrate (Bi(NO_3_)_3_·5H_2_O, AR), potassium chloride (NaCl, AR), mannitol (AR), polyvinylpyrrolidone (PVP, Mw = 58 000), and sodium borohydride (NaBH_4_, AR) were obtained from Shanghai Aladdin Biochemical Technology Co., Ltd. (Shanghai, China). All the chemicals were used without further purification. Bovine fibrinogen (Mw: 340 kDa) and lyophilizing thrombin powder were purchased from Yeasen Biotechnology (Shanghai) Co., Ltd. (Shanghai, China) and Hunan Yige Pharmaceutical Co., Ltd. (Tianjin), respectively.

### Synthesis of Bi/BiOCl Nano‐Heterojunction

Typically, 1.820 g mannitol was dissolved in deionized water. Then, 1.944 g Bi(NO_3_)_3_·5H_2_O was added to the above solution. After 30 min of vigorous stirring, 10 mL of NaCl saturated solution was added dropwise into the mixture with stirring and a uniform white suspension was formed. After another 10 min of agitation, the mixture solution was transferred into a 100 mL Teflon‐lined stainless steel autoclave, which was then heated up to 160 °C, maintained for 3 h, and then, cooled down naturally. The resulting product was collected for the following experiment.

0.250 g of PVP was dissolved in 25 mL deionized water and 0.500 g of as‐synthesized BiOCl nanoplates was added to the above solution with 15 min magnetic stirring. Then, a certain concentration of NaBH_4_ (15 mL) was added quickly into the above mixture, followed by 10 min of magnetic stirring. After the reaction, the products were collected by centrifugation and washed with anhydrous ethanol and deionized water, respectively, in sequence three times, and finally dried under vacuum. When the final concentrations of NaBH_4_ were 25, 50, and 100 mmol L^−1^, the obtained samples were denoted as 25Bi/BiOCl, 50Bi/BiOCl, and 100Bi/BiOCl. Unless otherwise specified, the Bi/BiOCl was 100Bi/BiOCl.

### Synthesis of Bi/BiOCl Nanojunction Composite Gel

Fibrin gels were synthesized through spraying equal volumes of thrombin (10 mg mL^−1^) and fibrinogen (50 U mL^−1^) with Bi/BiOCl. The obtained hydrogels were named as Bi/BiOCl@Gel‐*X* (BBFG‐*X*), where *X* was the mass percent concentration of Bi/BiOCl (in the text, 1, 3, and 7 stands for the concentration of Bi/BiOCl of 0.01, 0.03, and 0.07 mg mL^−1^, respectively). If *X* was not stated, the mass concentration of Bi/BiOCl was 0.07 mg mL^−1^.

### General Characterization of Bi/BiOCl and Composite Gel

The morphology of the samples was characterized by scanning electron microscope (SEM, Merlin, Zeiss) and TEM (JEM‐2100F, JEOL, 200 kV). For SEM/TEM observation, the Bi/BiOCl was ultrasonically dispersed. The suspension was then dropped on the surface of the carbon copper grid and dried in the air. The structural and element composition of the BiOCl were characterized by XRD (Empyrean, Panaco Netherlands) and XPS (ThermoFisher ESCALAB XI+), calibrating all binding energies using impurity carbon (C 1s = 284.8 eV) as reference, respectively. The UV–vis absorption spectra were obtained on an ultraviolet–visible diffuse reflection (UV–vis DRS) (SHIMADZU UV‐3600 Plus) with a self‐supporting sample cell, and the pure BaSO_4_ was used as a reflectance standard. The photoluminescence spectra (PL) were obtained on the FLS980 (Edinburg, England). Photocurrent–time curves and electrochemical impedance spectroscopy (EIS) data were recorded by an electrochemical workstation (Zahner, Germany), wherein platinum was used as the counter electrode and the Ag/AgCl electrode served as the reference electrode. A NIR laser of 808 nm was used as a light irradiation source. Na_2_SO_4_ solution (0.5 mol L^−1^) served as the electrolyte. The samples were immersed in 200  µL phosphate‐buffered saline (PBS) to facilitate equilibrium during light irradiation under the aforementioned power. The surface temperature of samples was recorded at 1 min intervals for a total of 10 min by a Fotric‐220 infrared imaging camera (Fotric, Germany). The heating–cooling cycle curve was obtained under 808 nm irradiation.

The ROS sensor, 2′,7′‐dichlorodihydrofluorescein (DCFH), was used to determine the in vitro ROS generation ability. All tests were performed in a dark environment to eliminate the influence of light. The samples immersed in 100 µL DCFH solution in 96‐well plates were obtained and subjected to 808 nm. The dye was consumed every 3 min to determine the ROS content. Electron spin resonance (ESR) and spin trapping methods were utilized to demonstrate the production of ROS. Equivalent quenching agents, including a 0.2 mol L^−1^ DMPO aqueous solution, a 0.2 mol mL^−1^ DMPO methanol solution, and a 5 mg mL^−1^ TEMP aqueous solution, were employed to detect signals of hydroxyl radicals, superoxide radicals, and singlet oxygen, respectively. Following a 5‐min irradiation period, equivalent quenching agents containing 500 µg mL^−1^ powder were introduced into the quartz capillary, and the ESR spectra were recorded using an EMXPlus‐10/12 instrument (Bruker, Japan).

Each hydrogel was lyophilized after freezing at −80 °C under vacuum for 48 h. After the dried hydrogels were coated with platinum, they were inspected under SEM. The rheological property of each hydrogel was recorded in 0.1–10% frequency sweep mode at 37 °C by a rotary rheometer (Physician MCR301, Anton Paar).

### Theoretical Simulation

All the calculations were performed in the framework of the density functional theory with the projector augmented plane‐wave method, as implemented in the Vienna ab initio simulation package.^[^ ^38^ ^]^ The generalized gradient approximation proposed by Perdew–Burke–Ernzerhof (PBE) was selected for the exchange‐correlation potential.^[^ ^39^ ^]^ The cut‐off energy for the plane wave was set to 450 eV. The energy criterion was set to 10^−5^ eV in the iterative solution of the Kohn–Sham equation. All the structures were relaxed until the residual forces on the atoms had declined to less than 0.02 eV Å^−1^. To avoid interlaminar interactions, a vacuum spacing of 20 Å was applied perpendicular to the slab. The formation energy *E*_form_ was expressed as:

(1) $$\;E_{\mathrm{form}}=E_{\mathrm{defect}}\;-E_{\mathrm{perfect}}-E_{\mathrm{Fe}}$$

where *E*_defect_ and *E*_perfect_ were the total energy of the defect and perfect model, respectively. *E*_Fe_ was the chemical potential of the Fe atom.

The electromagnetic field on the surface of Bi/BiOCl was calculated using COMSOL Multiphysics. This calculation allowed for the evaluation of the stimulated resonance oscillation of valence electrons, which led to a reduction in reflected light intensity and the generation of localized surface plasmon resonance through resonance energy transfer between the evanescent wave and surface plasmons.

### Antibacterial Activity

Considering that *E. coli*, ATCC25922 is the typical Gram‐negative bacteria and *S. aureus*, ATCC25923 is the typical Gram‐positive bacteria, the antibacterial activity of Bi/BiOCl against these two types of bacteria was observed. Bacteria were incubated in LB medium for 24 h at 37 °C, and then, mixed with powder sample (500 µg mL^−1^) at a density of 1 × 10^6^ CFU mL^−1^. For irradiation group, the bacteria received 10‐min irradiation treatment by 808 nm laser at a power density of 1.5 W cm^−2^. The mixture was taken to dilute 1 × 10^2^ times. The diluted 50 µL mixture was spread on each LB agar plate, and then, incubated at 37 °C for 12 h to determine the number of survival cells (in CFU). The bacterial suspension (1 × 10^6^ CFU mL^−1^) without photocatalyst and irradiation was used as a control group. The antibacterial performance was assessed as the equation: Antibacterial efficiency (%) = (1*−N*_experiment_ */N*_control_) × 100, where *N*_control_ was the count of CFU in the control group, and *N*_experiment_ was the count of CFU in the experiment group. The 100 µL bacterial suspension (1 × 10^6^ CFU mL^−1^) was dropped on the surface of gel. The antibacterial experiments of composite gels were similar to those of Bi/BiOCl heterojunctions. Antibacterial performance was also evaluated by live/dead fluorescence staining. Living *S. aureus* stain green whereas dead ones stain red. Bacterial morphology was observed by SEM. *S. aureus* and samples were co‐cultured with and without 808 nm irradiation. After being fixed with 2.5% glutaraldehyde solution for 2 h, the samples were sequentially dehydrated with 30%, 50%, 70%, 90%, and 100% ethanol for 20 min, respectively. After being dried, the sample was coated with platinum in a sputter coater and observed by SEM.

### Cell Experiments

HUVEC cells were seeded on the surface of samples in 48‐well plates (5 × 10^4^ cells per well) and cultured for 1 and 3 days. The cell viability was determined using the Cell Counting KIT‐8 (CCK‐8, Dojindo, Japan) assay, and the optical density (OD) value at 450 nm was obtained using a microplate reader (Cytation 5, BioTek, USA.). Live/dead assay of HUVECs was performed using Calcein‐AM/Propidium iodide (CalceinAM/PI, Invitrogen) staining. After the HUVECs were double‐stained with CalceinAM/PI for 20 min, the viability of HUVECs was evaluated by an inverted fluorescence microscope (Zeiss Axio Observer 7, Germany). In vitro HUVECs migration assay followed and HUVECs were seeded into 24‐well plates at a density of 5 × 10^4^ per well. After overnight incubation, a straight scratch was created by pipette tip (1 mL), and the cells were gently washed twice with PBS. A Transwell containing Fibrin gel or Bi/BiOCl@Fibrin gel was placed in each well. After 48 h of culture, HUVECs were stained with propidium iodide. Afterward, the cells were washed with PBS twice and imaged using an inverted fluorescence microscope.

### Animal Experiments

Male BALB/c mice (6 weeks old) were purchased from the Laboratory Animal Centre of the Southern Medical University (Guangzhou, China). Briefly, streptozotocin (50 mg kg^−1^) was administered through i.p. injection for 5 consecutive days to establish the diabetic mouse model. The blood glucose of the mice was monitored using a glucometer (Haier blood glucose meter, China). Mice with blood glucose levels < 16.7 mm were excluded, and the rest were confirmed diabetic mice. Diabetic mice were anaesthetized by phenobarbital sodium, and a round full thickness wound (diameter: 7 mm) was made on the back of the mice using scissors. Fifty mL of *S. aureus* (1 × 10^8^ CFU mL^−1^) was then dropped onto the wound to induce diabetic skin infection. After 10 min, the infected wound was washed with a PBS solution twice. Immediately, fibrin gels containing different formulations (Gel, BB@Gel) were sprayed on to cover the wound via a dual‐cartridge sprayer. Meanwhile, the mice were divided into four groups: Control, Fibrin gel, Bi/BiOCl@Fibrin gel, and BBFG + NIR group. For Control, Bi/BiOCl@Fibrin gel, and BBFG+NIR group, the mice were irradiated with 808 nm, 1.5 W cm^−2^ for 10 min, and temperature changes were recorded with a thermal infrared imaging camera (Fotric 220, Germany). Untreated *S. aureus*‐infected mice served as controls. Wounds were photographed every 2 days, and the length and width were recorded to calculate the area. At day 4, sterile cotton swabs were used to collect bacteria from the wound bed. After dilution, the bacteria were evenly coated on the LB agar and cultured for counting. Representative mice in each group were sacrificed at 4, 8, and 14 days, and the wounded skin was collected for histological and immunofluorescent staining. All experimental procedures at the animal level were performed under the approval of the Animal Care Committee of South China University of Technology (approval number: 2022098).

### Statistical Analyses

Data analysis was conducted using Prism 8 software (GraphPad Software Inc., CA, USA). Results are expressed as mean ± standard deviation (SD). Statistical significance was determined using unpaired two‐tailed Student's *t*‐tests for comparisons between two groups and ANOVA tests with Dunnett's or Tukey's post‐test for multiple groups. Sample sizes (*n*) and data preprocessing normalization details are provided in the corresponding figure legends. Significance was set at *P* < 0.05. (*P < 0.05, **P < 0.01, and ***P < 0.001).

## Conflict of Interest

The authors declare no conflict of interest.

## Supporting information

Supporting Information

## Data Availability

The data that support the findings of this study are available from the corresponding author upon reasonable request.
