# Supplementary material for: Localized Surface Plasmon Resonance‐Enhanced Photocatalytic Antibacterial of In Situ Sprayed 0D/2D Heterojunction Composite Hydrogel for Treating Diabetic Wound
Source: Adv Healthc Mater. 2024 Aug 26;13(29):2303836. doi: 10.1002/adhm.202303836 (PMC11582506; doi:10.1002/adhm.202303836)
Supplement: Supplementary file 1 — Supporting Information [file ADHM-13-0-s001.docx]

Supporting Information

Localized Surface Plasmon Resonance-Enhanced Photocatalytic Antibacterial of In-situ Sprayed 0D/2D Heterojunction Composite Hydrogel for Treating Diabetic Wound

*Zhengao Wang, Wei Li, Youzhun Fan, Cairong Xiao, Zhifeng Shi, Yunbing Chang, Guoyan Liang, Chengli Liu, Zurong Zhu, Peng Yu*, Xuebin Yang*, Zhiguo Song* and Chengyun Ning*


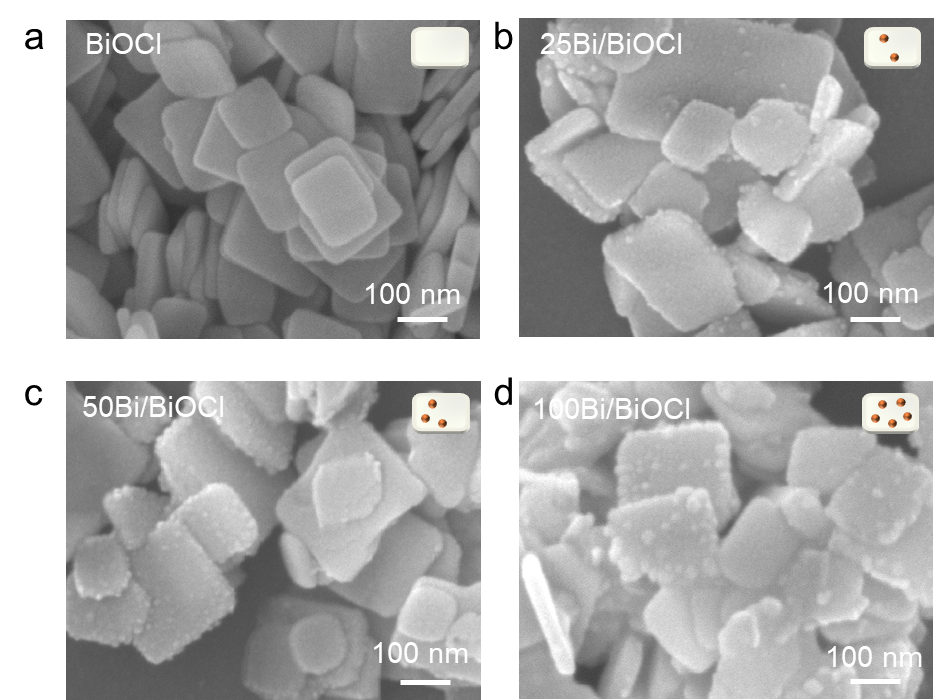


**Figure S1.** SEM images of the BiOCl (a), 25Bi/BiOCl (b), 50Bi/BiOCl (c) and 100 Bi/BiOCl (d).


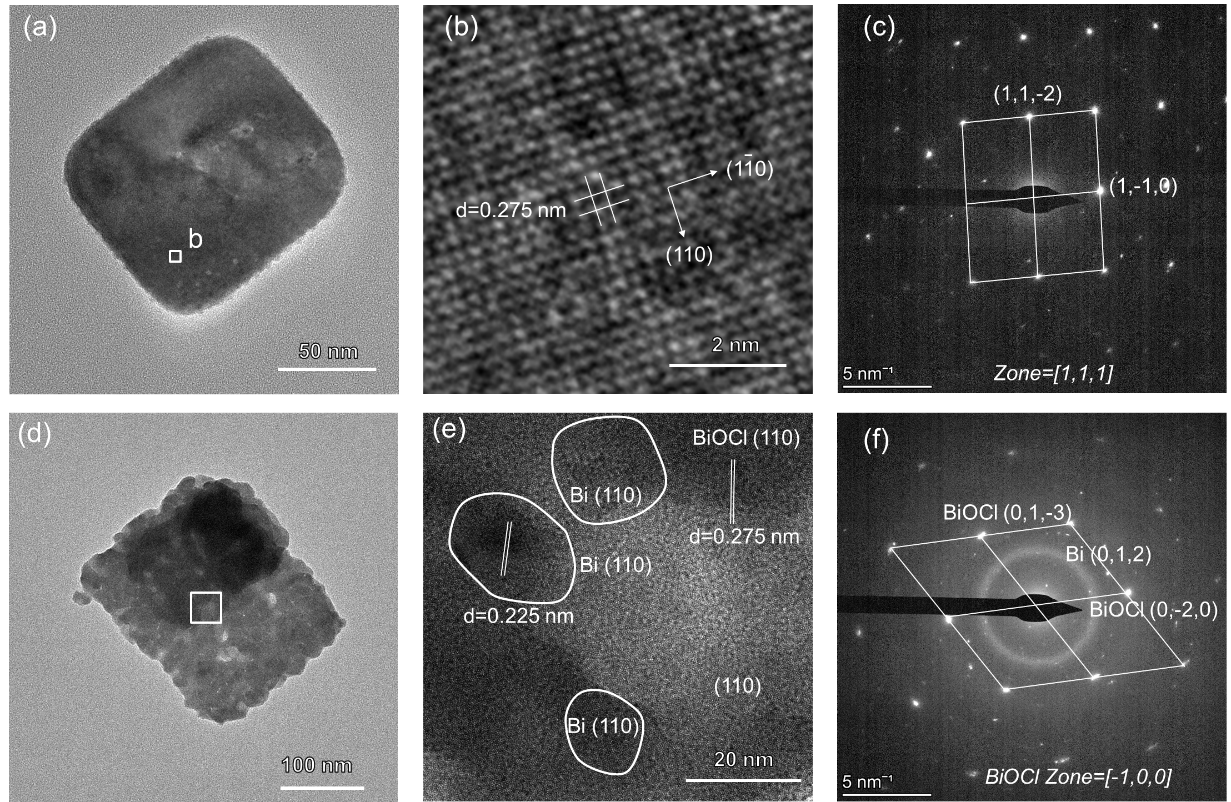


**Figure S2.** TEM images (a), HRTEM (b) images and SAED patterns (c) of pure BiOC, TEM images (d), HRTEM images (e) and SAED patterns (f) Bi/BiOCl.


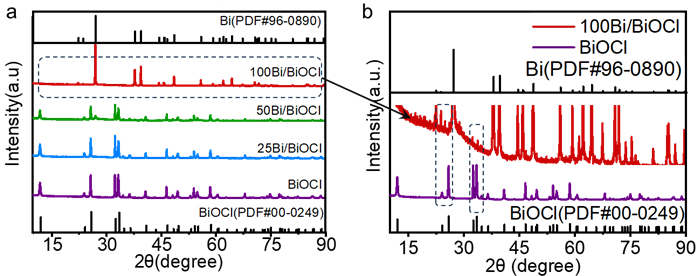


Fiuge S3. (a) XRD patterns of pure BiOCl and Bi/BiOCl. (b) partial magnified detail of 100Bi/BiOCl.


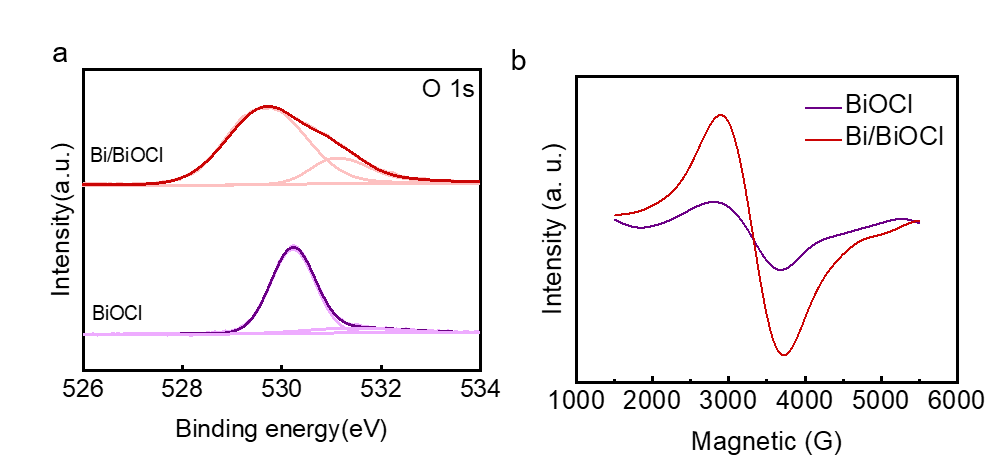


Figure S4. (a) XPS narrow scan for O. (b) ESR spectra of the pure BiOCl and Bi/BiOCl sample.


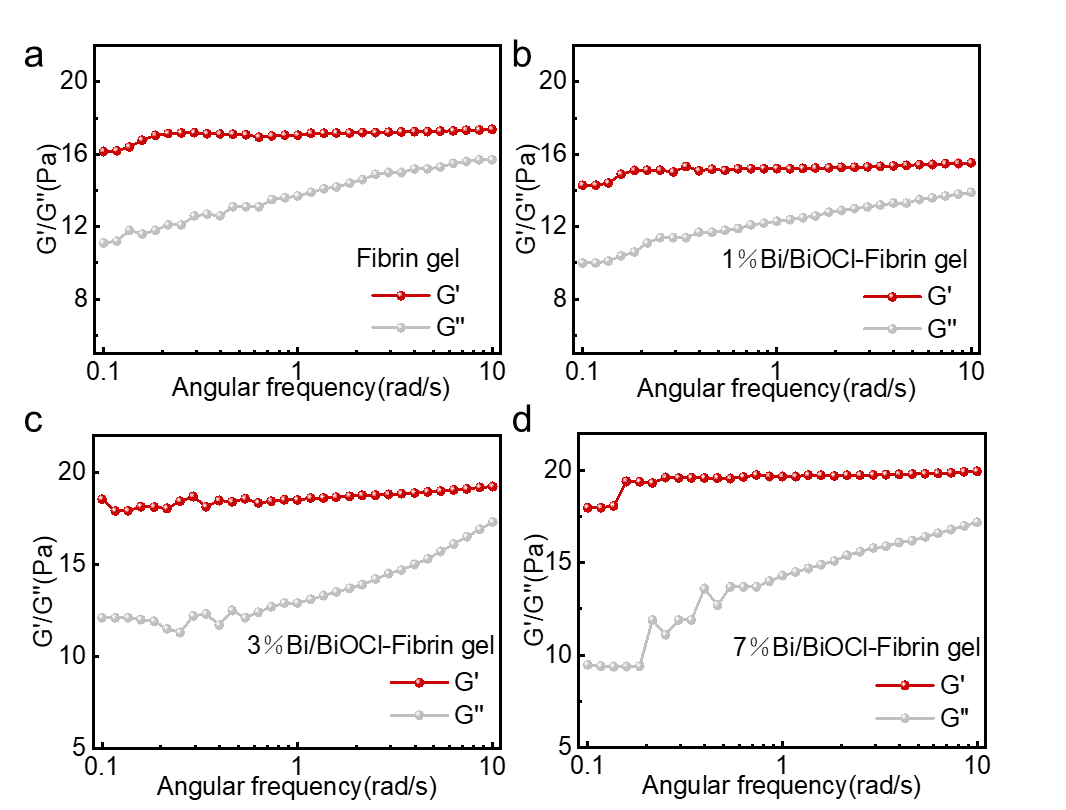


**Figure S5.** Rheological properties of Fibrin gel (a) and Bi/BiOCl@Fibrin gel (b, c, d).


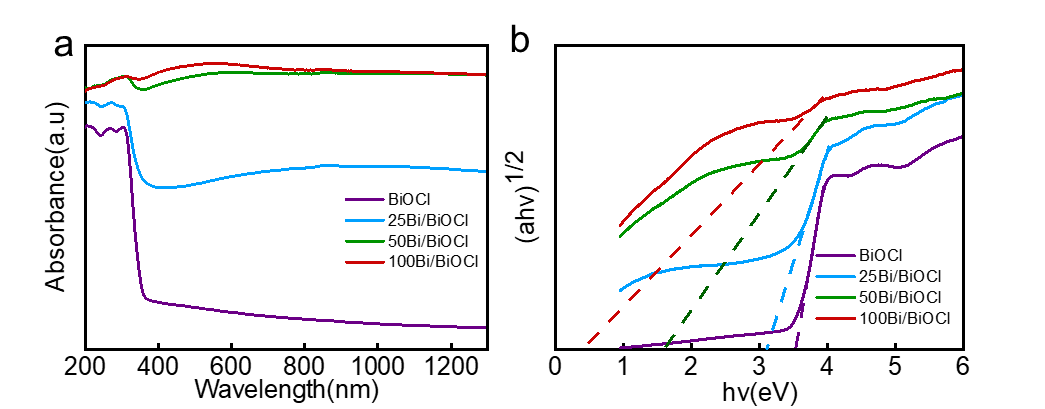


**Figure S6.** (a) UV–vis absorption spectra and (b) Band gap energy of the BiOCl, 25Bi/BiOCl, 50Bi/BiOCl and 100 Bi/BiOCl.


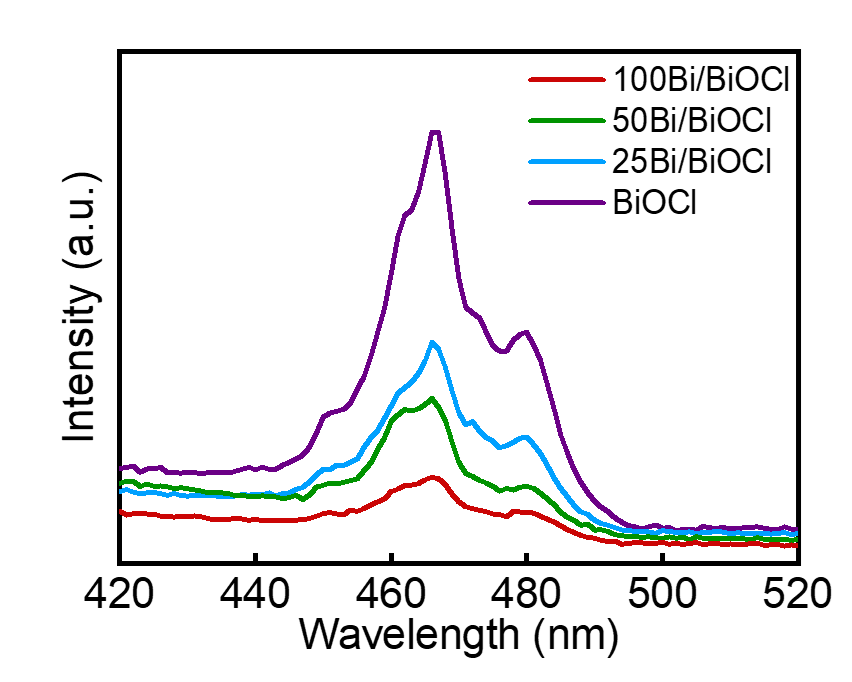


**Figure S7.** PL spectrum Bi/BiOCl with various with Bi nanoparticles of various densities


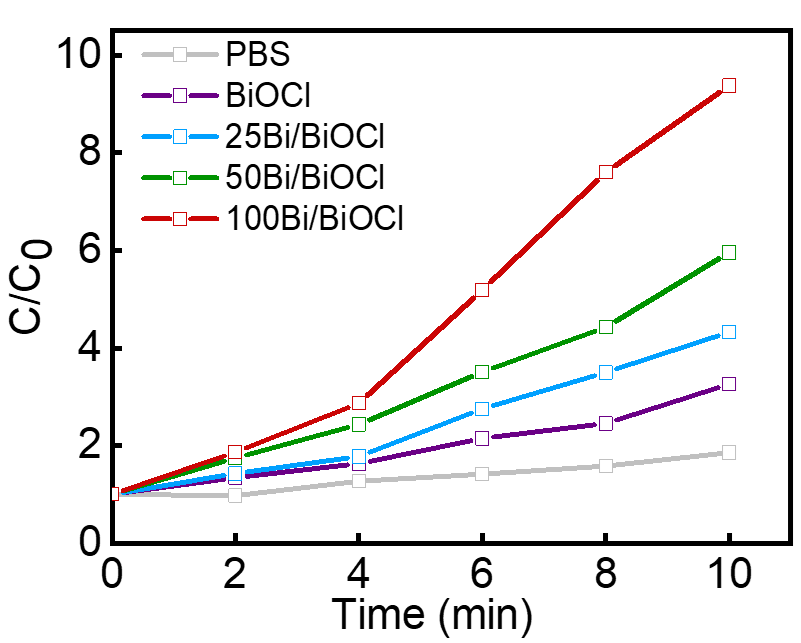


**Figure S8.** Bi/BiOCl-induced ROS production


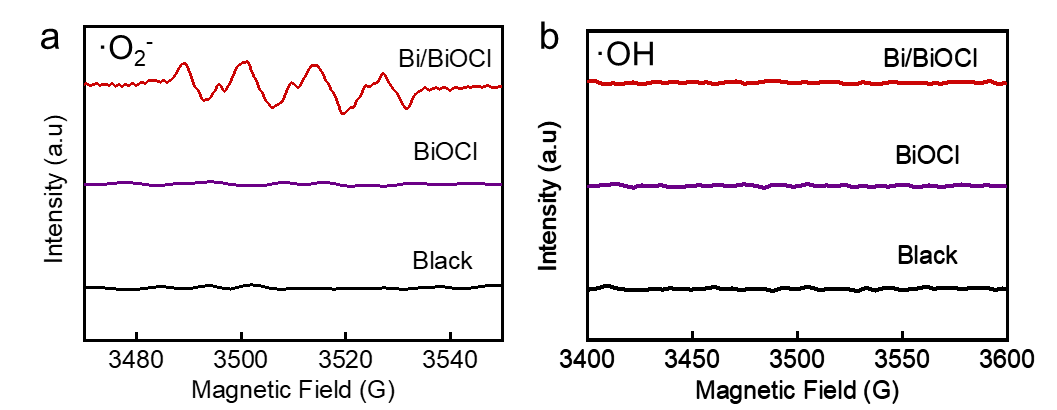


**Figure S9.** ESR spectra of radical adducts in Bi/BiOCl heterojunction for (a) the superoxide radical in methanol dispersion with DMPO and (b) the hydroxyl radical (·OH) in DMPO aqueous solutions.


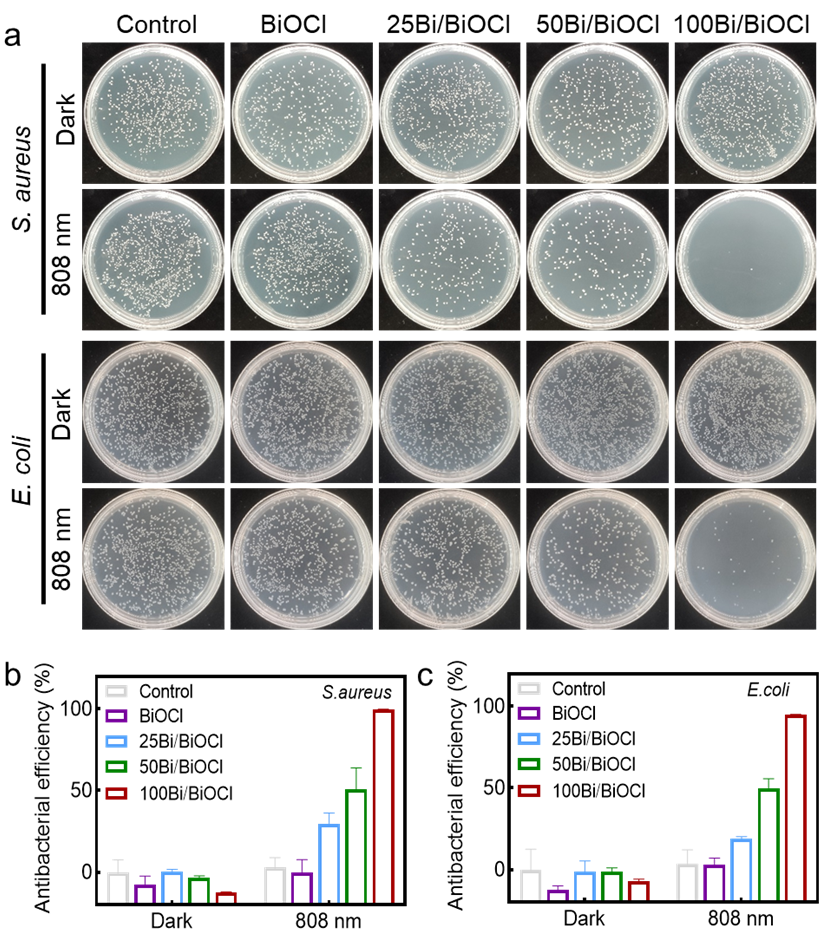


**Figure S10**. Antibacterial photos and antibacterial rates of Bi/BiOCl plates with/without 808 nm near-infrared light irradiation. (a) Photos of *S. aureus* and *E. coli colonies*. Antibacterial dfficiency of Bi/BiOCl against *S. aureus* (b) *E. coli* (c).


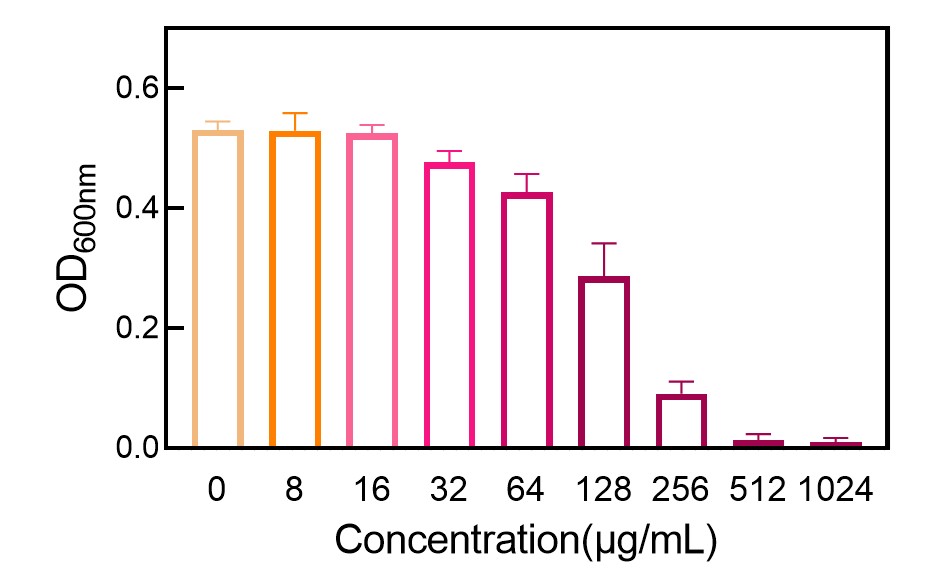


Figure S11. The minimum inhibitory concentrations of photoresponsive Bi/BiOCl against MRSA evaluated by the two-fold serial dilution method.


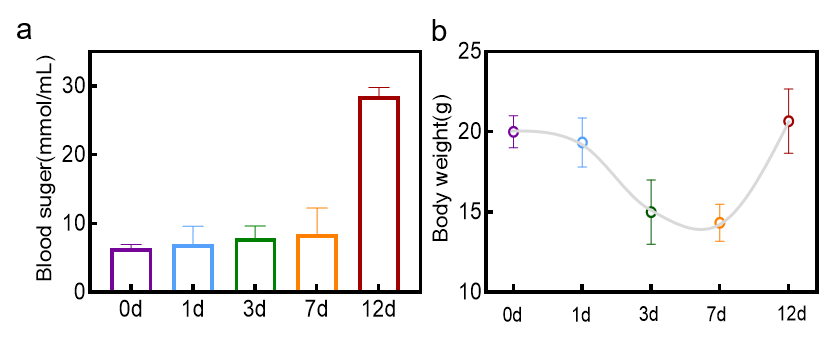


**Figure S12.** Changes in blood glucose (a) and body weight (b) in male BALB/c mice with STZ treatment.

**Figure S13.** antibacterial ratio of S. aureus from the wounds of different groups cultured. Data represents Mean +/- SD. n=6. ns, not statistically significant. ***p<0.001.
